# Apigenin attenuates atherosclerosis and non-alcoholic fatty liver disease through inhibition of NLRP3 inflammasome in mice

**DOI:** 10.1038/s41598-023-34654-2

**Published:** 2023-05-17

**Authors:** Zheng Lu, Lu Liu, Shunxin Zhao, Jiangtao Zhao, Sujun Li, Mingyang Li

**Affiliations:** 1 Medical Department of Henan Provincial People’s Hospital, Zhengzhou, Henan China; 2 Department of Nephrology, The First Affiliated Hospital of Zhengzhou University, Zhengzhou, Henan China; 3 Department of Respiratory Medicine, The First Affiliated Hospital of Zhengzhou University, Zhengzhou, Henan China; 4 Department of Cardiology, The First Affiliated Hospital of Zhengzhou University, Zhengzhou, Henan China; 5 Department of Geriatric Endocrinology, The First Affiliated Hospital of Zhengzhou University, Zhengzhou, Henan China; 6 Department of Emergency, Henan Provincial People’s Hospital, Zhengzhou, Henan China

**Keywords:** Physiology, Medical research, Molecular medicine

## Abstract

Apigenin (APN), a flavone found in several plant foods with various biological properties such as anti-obesity, anti-inflammation and other abilities, alleviates atherosclerosis and non-alcoholic fatty liver disease (NAFLD) induced by a high fat diet (HFD) in mice. However, the underlying mechanisms have not been fully understood. In this study, we investigated the role of NLRP3 in anti-atherosclerosis and anti-NAFLD effect of APN in mouse models with NLRP3 deficiency. Atherosclerosis and NAFLD models were established by treatment of low density lipoprotein receptor-deficient (*Ldlr*^*−/−*^) mice and *NLRP3*^*−/−*^* Ldlr*^*−/−*^ mice with a HFD diet (20% fat and 0.5% cholesterol) with or without APN. *En face* lipid accumulation analysis, plasma lipid levels, hepatic lipid accumulation and inflammation were analyzed and quantified. For in vitro experiments, HepG2 cells were stimulated by LPS plus oleic acid (OA) in the absence or presence of APN (50 μM). Lipid accumulation and the effect of APN on the NLRP3/NF-κB signaling pathway were investigated. APN administration partly reversed atherosclerosis and hepatic lipid accumulation, and decreased body weight and plasma lipid levels in *Ldlr*^*−/−*^ mice when fed a HFD. Compared with *Ldlr*^*−/−*^ mice, *NLRP3*^*−/−*^* Ldlr*^*−/−*^ mice showed more severe atherosclerosis and hepatic lipid accumulation. Treating the HepG2 cells with APN reduced lipid accumulation. APN also inhibited activation of the NLRP3/ NF-κB signaling pathway stimulated by OA together with LPS. Our results indicate that APN supplementation prevents atherosclerosis and NAFLD via NLRP3 inhibition in mice, and suggest that APN might be a potential therapeutic agent for the prevention of atherosclerosis and NAFLD.

## Introduction

Non-alcoholic fatty liver disease (NAFLD) is a common disease, and its incidence is increasing each year^1,2^. NAFLD is principally characterized by ectopic fat accumulation in hepatocytes caused by injurious factors other than alcohol. It is now widely accepted that many factors, including inflammation, mitochondrial dysfunction, oxidative stress, and adipose disorder, are involved in NAFLD progression^3–5^. NAFLD patients are at higher risk of cardiovascular diseases such as atherosclerosis, a chronic inflammatory artery disease with many metabolic changes^6^. Patients with NAFLD usually show many metabolic features similar to patients with atherosclerosis, such as dyslipidemia, obesity, inflammation and diabetes^7^. Studies have shown that inflammation plays a critical role in the initiation and progression of NAFLD and atherosclerosis^8,9^.

The inflammasome NLRP3 is a multiprotein scaffold that can mediate the activation of inflammatory reactions (10, 11). Activation of NLRP3 triggers local and systemic inflammation and has been linked to the pathogenesis of NAFLD and atherosclerosis^10–12^. NF-κB is the critical transcriptional factor for the activation of NLRP3 inflammasome and subsequent upregulation of the transcription of inflammatory cytokines such as tumor necrosis factor-α (TNF-α), interleukin-1β (IL-1β), and IL-18^13^. Many studies have reported that inhibition of NLRP3 inflammasome alleviates hepatic inflammation and fibrosis in mice^14–16^. Therefore, exploring inhibitors of the inflammatory regulators such as NLRP3 might represent a promising strategy for the treatment of NAFLD and atherosclerosis.

Apigenin (APN) is a natural bioflavonoid and is abundant in fruits, vegetables, herbs and spices^17^. APN has been shown to exert diverse pharmacological activities such as anti-inflammatory activity^18^, antioxidant^19^, and antitumor activities^20^. Moreover, many studies have demonstrated that APN ameliorates diabetes^21^, obesity^22^, atherosclerosis^23^, and hepatic lipid accumulation^24,25^ in mice. However, the effects of APN on NAFLD and atherosclerosis and the underlying mechanism have been poorly explored.

Therefore, in our study, we investigated the possible preventive effect of APN on NAFLD and atherosclerosis in vivo, using low density lipoprotein receptor-deficient (*Ldlr*^*−/−*^) mice and *NLRP3*^*−/−*^*Ldlr*^*−/−*^ mice fed a high-fat diet (HFD). And in vitro experiment, HepG2 cells were stimulated by LPS plus oleic acid (OA). Our findings demonstrates that APN has beneficial effects on NAFLD and atherosclerosis. The possible mechanism may involve the inhibition of NLRP3/NF-κB signaling pathway.

## Materials and methods

### Animals

Both *Ldlr*^*−/−*^ mice and *NLRP3*^*−/−*^ mice were purchased from Cyagen Laboratories. *Ldlr*^*−/−*^ mice on a C57BL/6 J background were crossed with *NLRP3*^*−/−*^ mice to generate *NLRP3*^*−/−*^*Ldlr*^*−/−*^ mice. Diet used in the study included a standard chow diet and a high-fat diet (20% fat and 0.5% cholesterol) purchased from Jiangsu Xietong Pharmaceutical Bioengineering Co. Ltd. Male *Ldlr*^*−/−*^ mice (8 weeks old, n = 8) were randomly divided into four groups, including chow diet (CD) group, 50 mg/kg/day apigenin (APN 50) group, high-fat diet (HFD) group, HFD + 5 mg/kg/day apigenin (HFD + APN 5) group, and HFD + 50 mg/kg/day apigenin (HFD + APN 50) group. These mice were kept on CD or HFD for 8 weeks. Male *NLRP3*^*−/−*^*Ldlr*^*−/−*^ mice (8 weeks old, n = 16) were randomly divided into two groups and treated with HFD with or without APN (50 mg/kg/day). APN was administrated by gavage every day. All of the mice were housed in pathogen-free and standard conditions (12:12-h light-dark cycle, a relative humidity of 60%, and room temperature of 22 °C) and had free access to water and food. All animal work was approved by the Animal Care Ethics Committee of the Henan Provincial People’s Hospital and was carried out in accordance with the Animals Act 1986, the National Institutes of Health Laboratory Animal Application Guidelines and the Regulations for the Administration of Affairs Concerning Experimental Animals published by the State Science and Technology Commission of China, and the ARRIVE guidelines.

### Blood metabolic analysis

Blood was obtained by retro-orbital bleeding. Plasma total cholesterol (TC) and triglyceride (TG) concentrations were determined by enzymatic methods (Sigma Kits, USA).

### Atherosclerotic lesion analysis

The aortas from the origin at the hearts were removed from the mice and fixed with 4% paraformaldehyde. The proximal aortas were embedded in OCT medium, and 4 μm-thick sections were prepared. Then, aortic sinus sections were stained using Oil Red O. The whole aortas were cut longitudinally and stained with Oil Red O. The lesion areas of the aorta and the aortic sinus were analyzed by using ImageJ software.

### Histological analysis

The livers were fixed in 10% formalin, embedded in paraffin, and then cut into 5 μm serial sections. The tissue sections were subjected to standard hematoxylin-eosin (H&E) staining for the determination of hepatic fat accumulation. OCT-embedded frozen livers were sectioned at 7 μm for Oil Red O staining.

*ELISA.* IL-1β and IL-18 levels in aortas were measured using commercial ELISA kits (cat# PMLB00C & DY122-05, Minneapolis, USA) in accordance with the manufacturer’s instructions.

### Immunohistochemistry staining

Macrophage contents in atherosclerotic lesions were measured using immunohistochemistry staining. Briefly, frozen aortic sinus sections were incubated with 3% H_2_O_2_ for 10 min, blocked with 3% BSA (Siama) for 1 h, and incubated with anti-F4/80 antibody (1:200, Abcam, Inc., CA, USA; Cat.No. ab-300421) overnight at 4 °C. After incubation with anti-rabbit lgG for 1 h at room temperature, the slides were developed with 3,3-diaminobenzidine (DAB Quanto Kit, TA-060-QHDX, ThermoFisher) and stained with hematoxylin. Images were recorded uding a light microscope.

### Cell culture

Human liver cancer lines, HepG2 (JCRB1054, lot no. 04202017) were purchased from JCRB Cell Bank. HepG2 were cultured in DMEM supplemented with 15% FBS and 1% penicillin-streptomycin in a humidified atmosphere of 5% CO2 and 95% air at 37 °C. The HepG2 cells used in the present study were regularly authenticated via morphologic observation and tested for the absence of mycoplasma contamination. Mycoplasma testing was performed using a MycoProbe® Mycoplasma Detection kit (R & D System, Inc) in accordance with the manufacturer’s protocol. The cells were starved in serum-free DMEM for 12 h followed by the incubation with OA for additional 24 h in the absence or presence of APN (25 and 50 μM).

### Cell viability assays

For cell viability, the cells were cultured at a density of 4–5 × 10^4^ cells per well in 96-well plates for 24 h. The cells were treated with different concentrations of APN for 24 h. Then cell viability was determined by the MTT reduction assay. The cells were incubated with MTT solution (5 mg/ml) for 4 h at 37 °C. The dark blue formazan crystals formed in intact cells were solubilized with 150 μl of DMSO, and the absorbance at 490 nm was measured with a microplate reader (Bio-Rad, Hercules, CA, USA).

### Western blotting

Total protein was extracted from cells and liver tissues using RIPA lysis buffer and phenylmethylsulfonyl fluoride (Beyotime, China). The protein concentration was detected by using a BCA protein assay kit. Equal amounts of protein (20 μg) were separated using 10% or 12% SDS-PAGE and were transferred onto polyvinylidene difluoride membranes (PVDF). Next, the PVDF membranes were blocked with 5% fat-free milk and incubated with primary antibodies to NLRP3 (Abcam, Inc., CA, USA; Cat. No. ab-270449), NF-κB/p-65 (Abcam, Inc., CA, USA; Cat. No. ab-76302), and GAPDH (Abcam, Inc., CA, USA; Cat. No.ab-181602) overnight at 4 °C. Subsequently, the membranes were washed and incubated with secondary antibodies at room temperature. The optical density of the bands was visualized by an ECL system (Pierce). GAPDH was used as an endogenous control. Data was normalized to GAPDH levels.

### RNA isolation and mRNA expression using reverse transcription-quantitative PCR (RT-qPCR)

Total RNA was extracted from the frozen tissues or treated cells using Trizol reagent (cat. No. 15596026; Invitrogen; Thermo Fisher Scientific, Inc.), as per the manufacturer’s protocol. First strand cDNA was synthesized using an RT kit (Invitrogen; Thermo Fisher Scientific, Inc.). qPCR was then performed using TB Green Premix Ex Taq II (Tli RNaseH Plus; cat. No.RR820A, Takara Bio, Inc.). The thermocycling conditions comprised an initial denaturation at 94 °C for 5 min, 40 cycles of 10 s at 94 °C and 20 s at 60 °C, and a final extension of 30 s at 72 °C. A single melting curve peak confirmed the presence of a single product. GAPDH was used as the reference control gene. Results were expressed as fold differences relative to GAPDH using the 2^-ΔΔCq^ method. All the primers were synthesized by Sangon Biotech (Shanghai, China) and the sequences are listed in Table 1.

**Table 1:** Primer list for quantitative real-time PCR.

Gene name	Forward primer (5'-3')	Reverse primer (5'-3')
F4/80	TTTCCTCGCCTGCTTCTTC	CCCCGTCTGTATTCAACC
TNFα	CTGTGAAGGGAATGAATGTT	CAGGGAAGAATCTGGAAAGGTC
MCP1	TCCCAATGAGTAGGCTGGA	AAGTGCTTGAGGTGGTTGT
IL-1β	AGGCTCCGAGATGAACAA	AAGGCATTAGAAACAGTCC
TGF-β1	GGCGGTGCTCGCTTTGTA	TCCCGAATGTCTGACGTAT
IL-6	TAGTCCTTCCTACCCCAATTTCC	TTGGTCCTTAGCCACTCCTTC
IL-18	GACTCTTGCGTCAACTTCAAGG	CAGGCTGTCTTTTGTCAACGA
NLRP3	ATTACCCGCCCGAGAAAGG	TCGCAGCAAAGATCCACACAG
NF-κB	ATGGCAGACGATGATCCCTAC	TGTTGACAGTGGTATTTCTGGTG
GAPDH	TCCTTGGAGGCCATGTGGGCCAT	TGATGACATCAAGAAGGTGGTGAAG

### Statistical analysis

All data are presented as means ± SEM. SPSS 21.0 was used to perform a statistical analysis of the data. Statistical differences were assessed with a two-tailed Student’s *t* test (for comparison of two conditions) and ANOVA(for comparison of more than two conditions). A *P* value lower than0.05 was considered statistically significant.

### Ethics approval

All of the animal work was approved by the Animal Care Ethics Committee of the Henan Provincial People’s Hospital and was carried out in accordance with the Animals Act 1986, the National Institutes of Health Laboratory Animal Application Guidelines and the Regulations for the Administration of Affairs Concerning Experimental Animals published by the State Science and Technology Commission of China, and the ARRIVE guidelines.

### Consent for publication

All the authors approved the publication.

## Results

### Apigenin ameliorates metabolic abnormalities in ***Ldlr***^***−/−***^ mice fed a HFD diet

**Figure 1:**
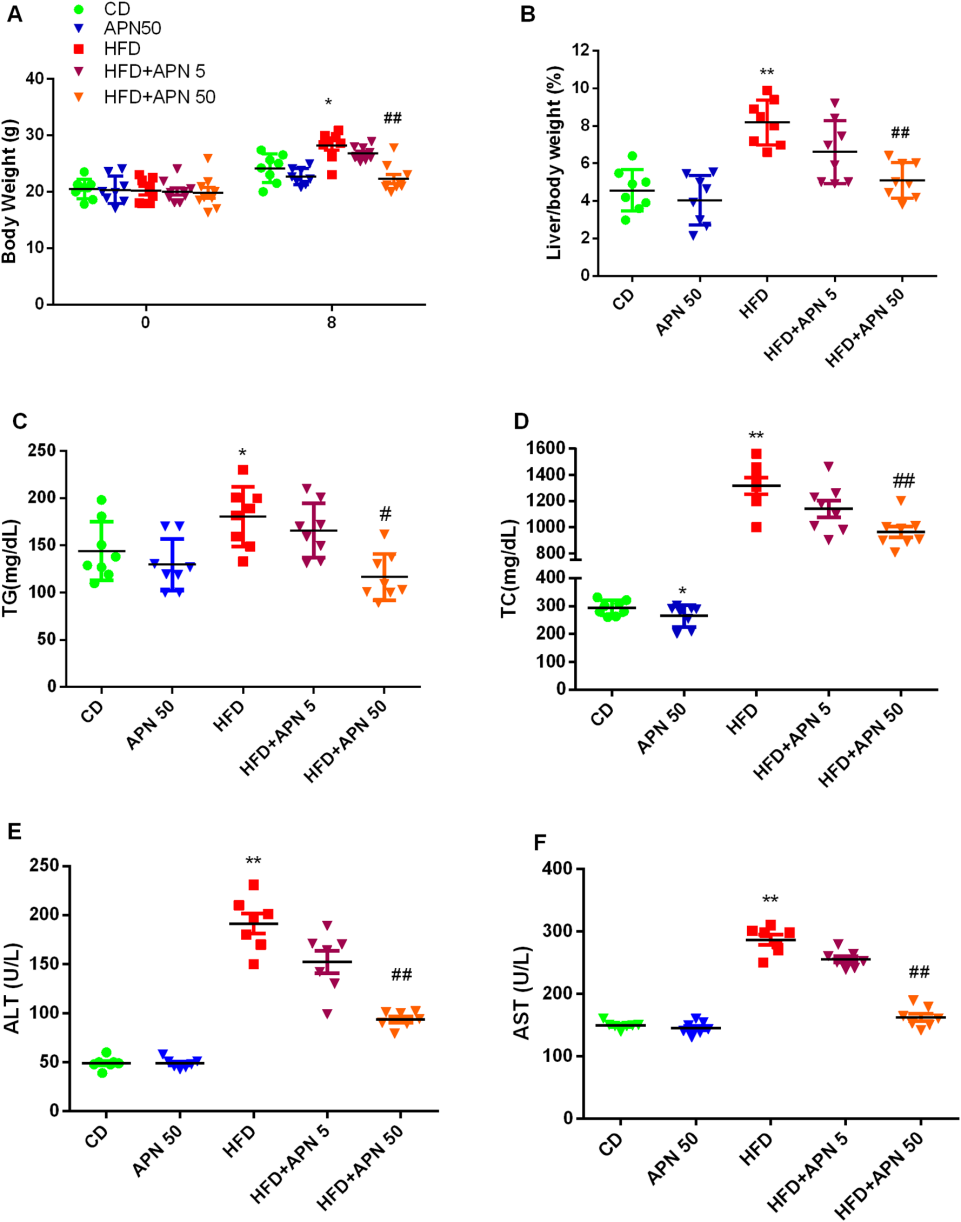
Effect of apigenin (APN) treatment on body weight, liver weight and plasma lipid levels of mice fed with a high-fat diet (HFD). Body weight (**A**), the ratio of liver weight to body weight (**B**), plasma TG (**C**), and TC (**D**) levels. Plasma levels of ALT (**E**) and AST (**F**). Data are presented as mean ± SEM, n = 8, *P < 0.05, **P < 0.01 versus CD mice. #*P* < 0.05, ^##^*P* < 0.01 versus HFD mice.

As expected, after treating the mice with a HFD diet for 8 weeks, a significant increase in body weight and liver weight were observed in the HFD group (Fig. 1A,B). In the apigenin-treated groups, the mice were given APN by gavage. Specifically, 50 mg/kg/day as the high-concentration group and 5 mg/kg/day as the low-concentration group were chosen for the in vivo experiments. APN treatment (50 mg/kg/day) significantly reversed the increase in body weight and liver weight as shown in Fig. 1A,B. Plasma total cholesterol (TC) and triglyceride (TG) levels were then analyzed. As shown in Fig. 1C,D, TG and TC levels in the HFD + APN 50 group were significantly lower than those in the HFD group. HFD in mice also induced hepatocyte damage as reflected by increased serum transaminases ALT and AST; however, APN 50 treatment significantly reduced the levels of ALT and AST in mice fed a HFD diet (Fig. 1E,F). These results demonstrated that the treatment with the high concentration (50 mg/kg/day) of APN could decrease liver injury in HFD-fed mice, but low concentration (5 mg/kg/day) of APN showed no obvious effect on liver injury. Therefore, the high concentration (50 mg/kg/day) of APN was chosen for the subsequent experiments.

### Apigenin attenuates liver steatosis induced by the HFD diet in *Ldlr*^*−/−*^ mice

**Figure 2:**
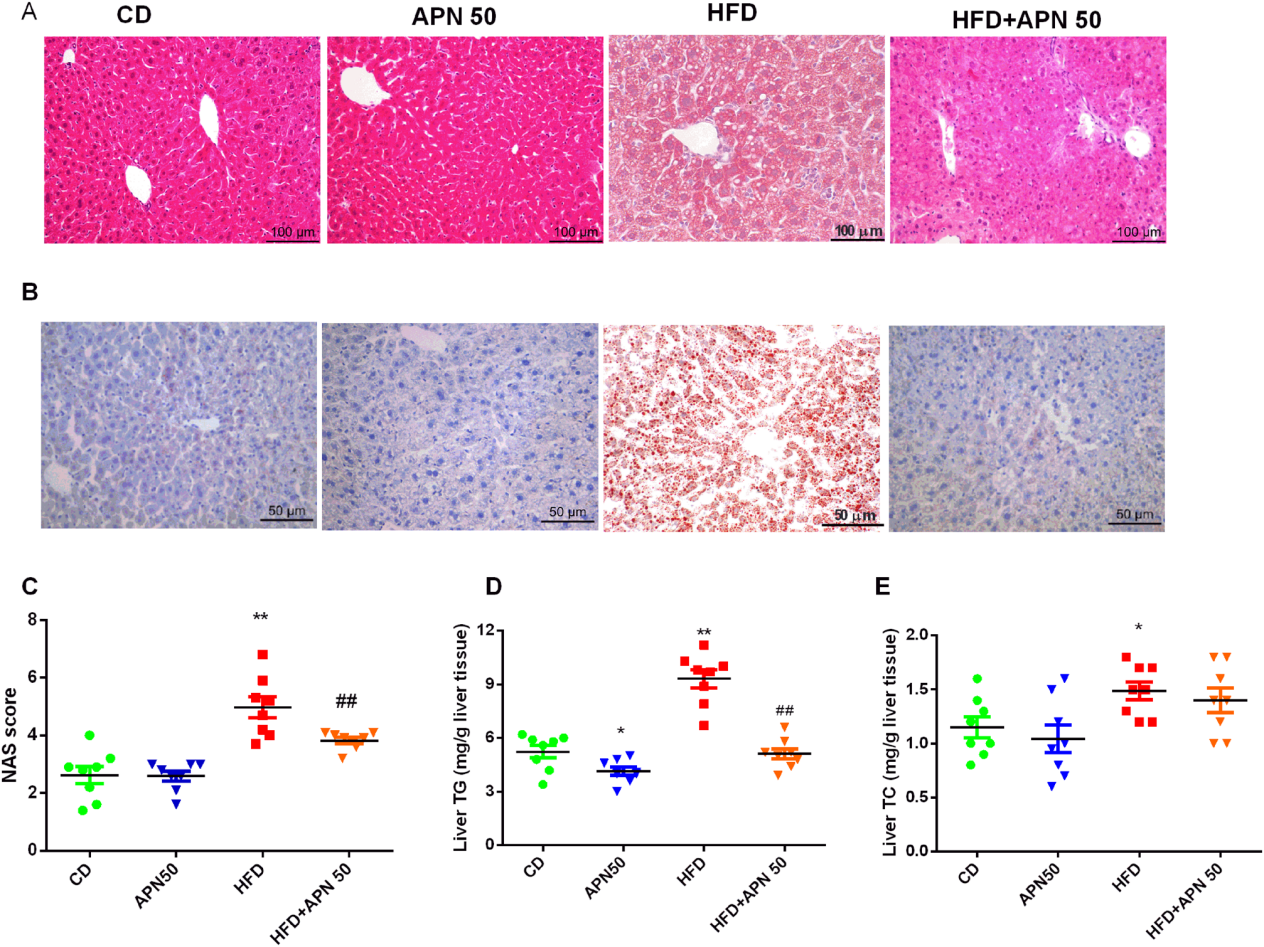
Effect of apigenin (APN) treatment on hepatic lipid accumulation in mice fed with a high-fat diet (HFD). Representative histology of H&E (**A**) and Oil Red O staining (**B**), NAS score (**C**), TG (**D**) and TC (**E**) in mice livers. Data are presented as mean ± SEM, n = 8, ^*^P < 0.05, ^**^P < 0.01 versus CD mice. ^##^*P* < 0.01 versus HFD mice.

We further analyzed hepatic lipid accumulation in the HFD and the APN treatment groups. The liver staining showed that the CD group had no lipid accumulation and HFD induced obvious hepatic lipid accumulation in the livers of the HFD group (Fig. 2A). Oil red O staining further confirmed this result (Fig. 2B). And, liver TG accumulation were increased by approximately a two-fold in the HFD group when compared with the CD group. This increase was significantly reduced after APN treatment (Fig. 2D). However, TC levels were not significantly changed in APN group mice compared with HFD group mice (Fig. 2E). The average NAS score of the liver was significantly decreased after APN treatment after HFD (Fig. 2C).

### Apigenin rduces inflammatory cytokines and alleviates atherosclerosis in HFD-fed *Ldlr*^*−/−*^ mice

**Figure 3:**
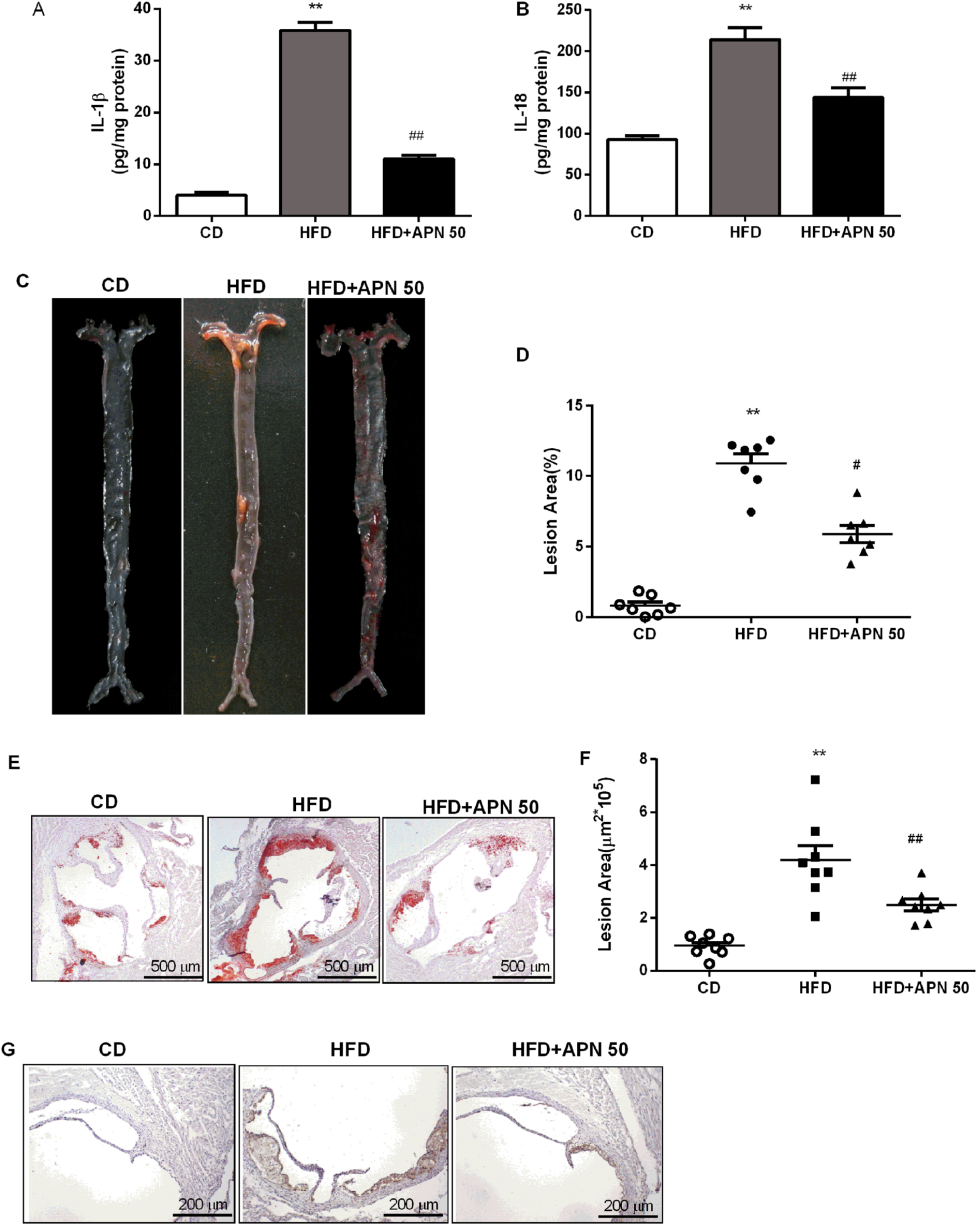
Apigenin attenuates inflammatory cytokines and alleviates atherosclerosis in HFD-fed *Ldlr*^*−/−*^ mice. **A** and **B**. IL-1β and IL-18 levels were measured by ELISA within aortas. **C** and **D**. Representative *en face* images of Oil Red O-stained aorta and quantification of lesion area. **E** and **F**. Representative aortic root sections stained with Oil Red O, and quantification of aortic lesion areas. **G**. Representative F4/80 immunostaining and quantification of aortic sinus lesions. Data are presented as the mean ± SEM, ^*^P < 0.05, ^**^P < 0.01 for HFD mice versus CD mice. ^#^*P* < 0.05, ^##^*P* < 0.01 for HFD mice versus HFD + APN 50 mice.

Inflammation is one of the main promoters of atherosclerosis. We then detected the levels of IL-1β and IL-18 in arteries with atherosclerosis. As shown in Fig. 3A,B, the levels of IL-1β and IL-18 were significantly elevated in arteries of *Ldlr*^*−/−*^ mice fed with a HFD, and APN 50 treatment decreased IL-1β and IL-18 levels in arteries. These results demonstrated that APN 50 reduced the production of inflammatory cytokines in atherosclerosis mice. Considering the alleviation of metabolic syndrome and decreased levels of proinflammatory cytokines after APN 50 treatment, we determined the effects of APN on atherosclerotic lesions. *En face* analysis of the aorta and quantification of Oil Red-stained cross sections of the aortic roots showed that atherosclerotic lesion sizes significantly decreased after APN 50 treatment (Fig. 3C–F). These results indicate that APN might have a protective role in atherosclerosis. In addition, macrophages play a key role in the development of atherosclerosis. Thus, we evaluated the effect of APN on macrophage accumulation in atherosclerotic lesions. As shown in immunohistochemical staining of the aortic sinus sections with macrophage antibody F4/80 (Fig. 3G), there were more F4/80 positive cells in the atherosclerotic lesions of the HFD group when compared with the control group, and APN 50 treatment significantly alleviated macrophage accumulation in atherosclerotic lesions. These findings demonstrated that APN not only attenuated atherosclerotic lesions but reduced macrophage accumulation in the aortic lesions.

### Apigenin protects against NAFLD through inhibiting the NLRP3/NF-κB signaling pathway

**Figure 4:**
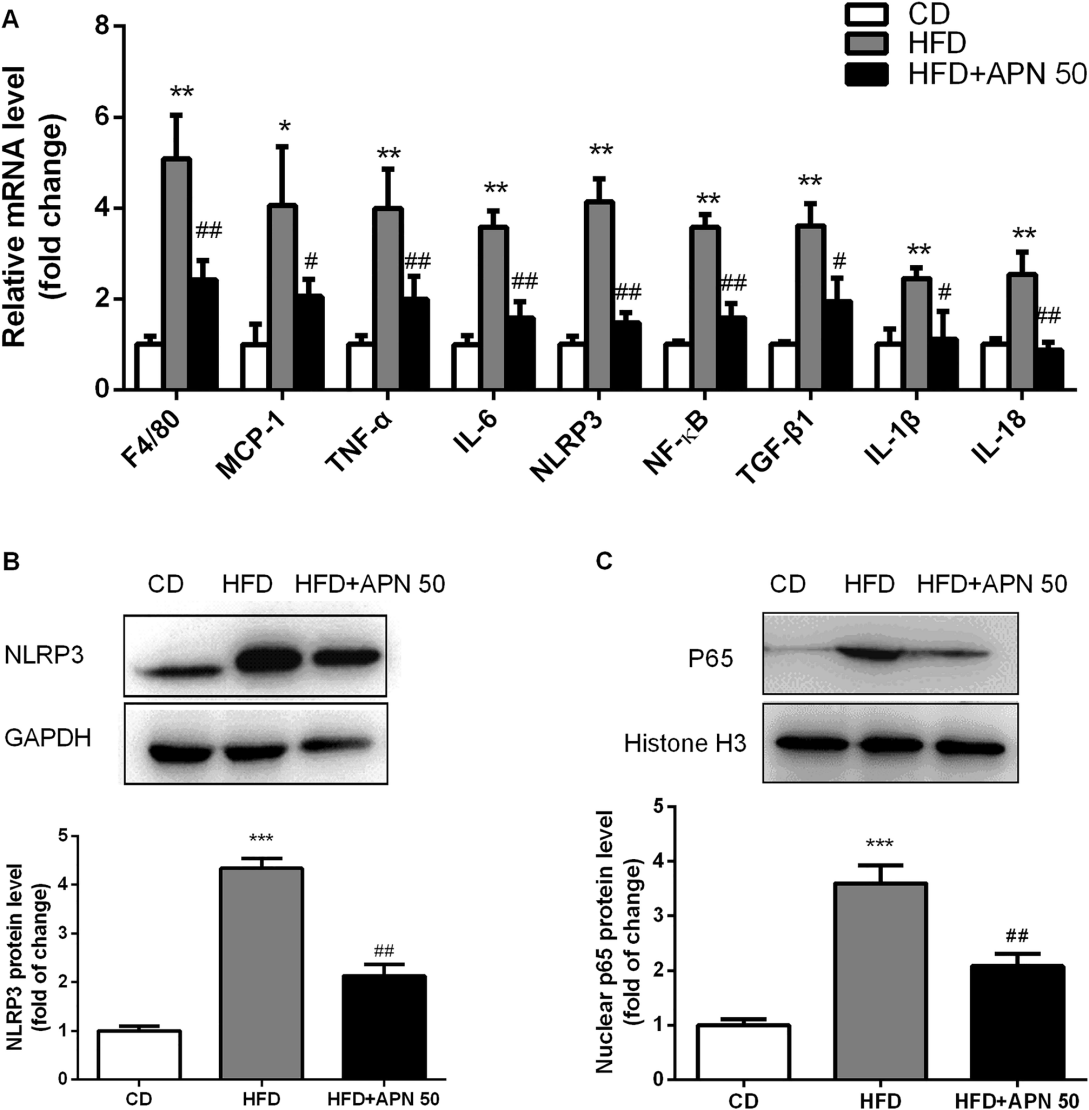
Apigenin protects against NAFLD by inhibiting the NLRP3/NF-κB signaling pathway A. The mRNA expression level of inflammatory genes in the livers of different groups of mice (n = 6 per group). B and C. Protein level and quantitative analysis of NLRP3 and NF-κB in different groups of mice. Data are presented as the mean ± SEM, ^*^P < 0.05, ^**^P < 0.01 for HFD mice versus CD mice. ^#^*P* < 0.05, ^##^*P* < 0.01 for HFD mice versus HFD + APN 50 mice.

Next, we examined inflammatory cytokine gene expression in the liver in each group of mice. As shown in Fig. 4A, HFD-fed mice had significantly increased mRNA levels of inflammatory genes (F4/80, MCP-1, TNF-α, IL-6, NLRP3, NF-κB, TGF-β1, IL-1β and IL-18), and APN 50 treatment was able to corrected the upregulated expression of these inflammatory genes. To explore novel mechanisms underlying the protective effects of APN on NAFLD, we focused on the NLRP3/NF-κB signaling pathway which has attracted a lot of attention in NAFLD progression. As shown in Fig. 4C, the hepatic protein level of nuclear NF-κB was significantly upregulated in the HFD mice, and APN 50 supplementation significantly reversed the HFD-induced increase of nuclear NF-κB protein level. Consistent with the change in nuclear NF-κB protein level, the hepatic protein expression of NLRP3 was significantly enhanced in the HFD group. And, APN administration greatly lower the hepatic level of NLRP3 as shown in Fig. 4B.

### Apigenin attenuates lipid accumulation via inhibiting the NLRP3/NF-κB signaling pathway in HepG2 cells

**Figure 5:**
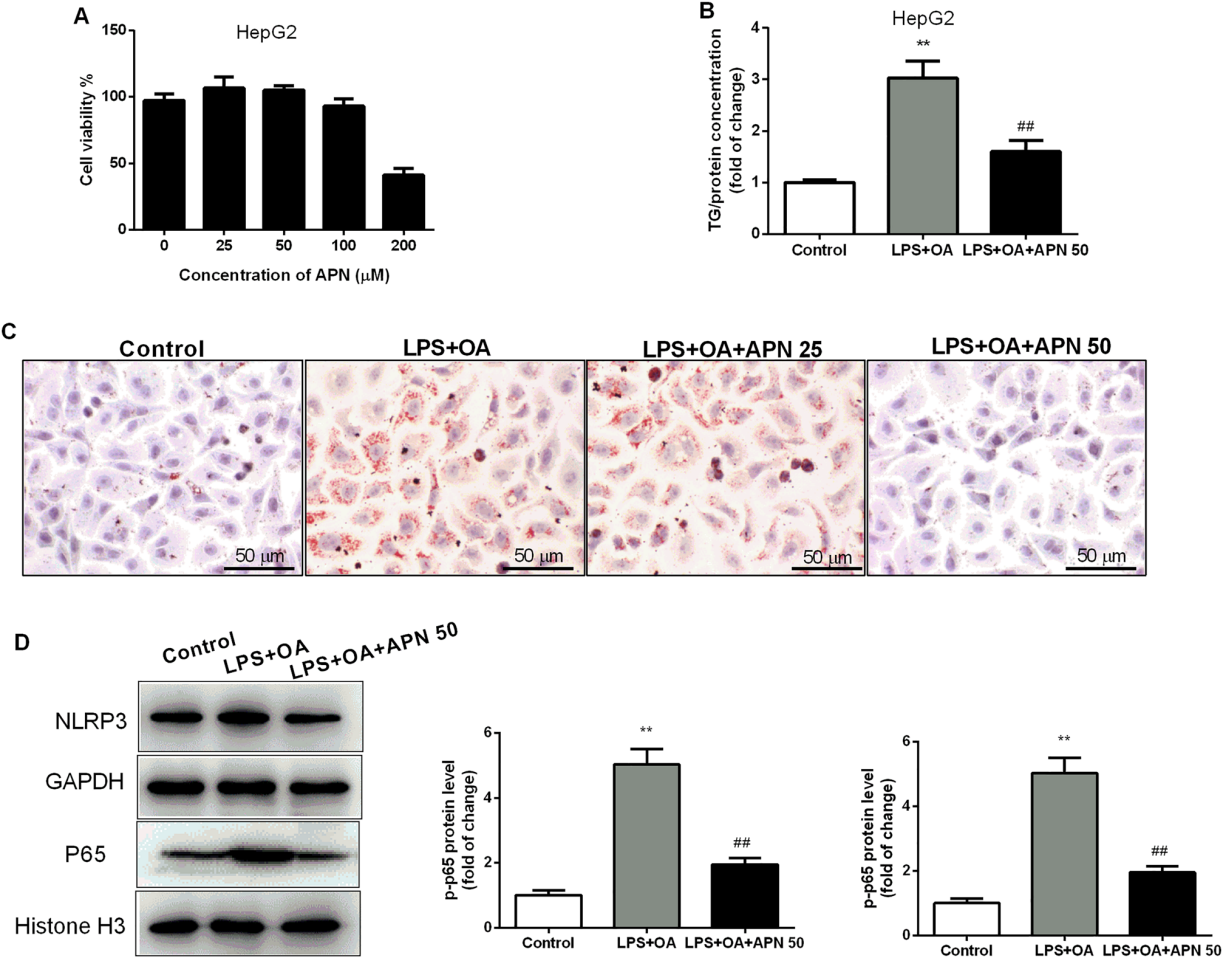
Apigenin ameliorates hepatocellular lipid accumulation in HepG2 cells stimulated by LPS and OA. (**A**). Cytotoxicity of apigenin in HepG2 cells. (**B**). TG contents in HepG2 cells stimulated by LPS and OA. (**C**). Representative histology of Oil Red O staining of HepG2 cells. (**D**), Protein level and quantitative analysis of NLRP3 and NF-κB in HepG2 cells stimulated by LPS and OA. Data are presented as the mean ± SEM, ^**^P < 0.01 for LPS + OA versus Control. ^##^*P* < 0.01 for LPS + OA versus LPS + OA + APN 50.

Furthermore, we established an in vitro NAFLD model in a fat overload profile in human liver cancer cells (HepG2 cells) by stimulation with LPS plus OA. First, we detected the cytotoxicity of APN on HepG2 cells (Supplementry Fig. 4). As shown in Fig. 5A, the results of the MTT assay showed that APN had no cytotoxic effects on HepG2 cells at concentrations less than 200 μM. Thus, in the following experiments, APN concentrations of 25 μM and 50 μM were chosen. HepG2 cells were cultured in medium containing LPS plus OA, and lipid accumulation in the cells was analyzed by Oil Red O staining. Treatment with 50 μM APN markedly decreased OA-induced lipid accumulation in HepG2 cells, as revealed by cellular triglyceride content and Oil red O staining (Fig. 5B,C). Consistent with the in vivo results, NLRP3 and NF-κB were activated after the stimulation of LPS plus OA in HepG2 cells, and treatment with 50 μM APN significantly reduced NLRP3 and NF-κB activation (Fig. 5D).

### NLRP3 deficiency impairs the anti-NAFLD and anti-atherosclerosis effects of APN

**Figure 6:**
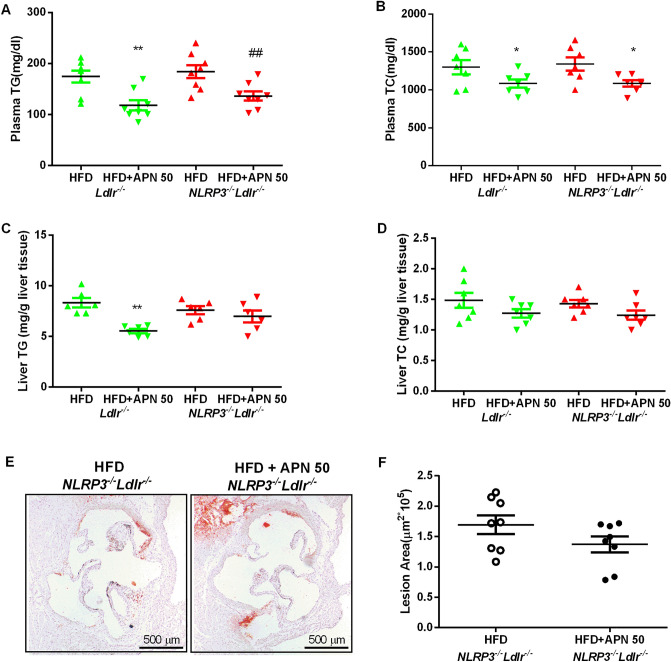
Apigenin has no effect on attenuation of hepatic lipid accumulation and atherosclerosis in *NLRP3*^*−/−*^*Ldlr*^*−/−*^ mice fed a high-fat diet (HFD). (**A**) and (**B)**. Plasma TG and TC levels in mice. (**C**) and (**D**). The content of TG and TC in mice livers. (**E**) and (**F**). Representative aortic root sections stained with Oil Red O, and quantification of aortic lesion areas. Data are presented as the mean ± SEM, ^*^P < 0.05, ^**^P < 0.01 for HFD mice versus HFD + APN 50 mice.

To further investigate the role of NLRP3 in the anti-NAFLD and anti-atherosclerosis effects of APN, *NLRP3*^*−/−*^*Ldlr*^*−/−*^ mice were generated and fed with HFD with or without APN 50 for 8 weeks. Plasma lipid levels were determined and showed an obvious decrease (Fig. 6A,B) between the HFD and HFD + APN 50 group in *NLRP3*^*−/−*^*Ldlr*^*−/−*^ mice. Liver weight and body weight did not significantly differ between *NLRP3*^*−/−*^*Ldlr*^*−/−*^ mice fed with HFD with and without APN (data not shown). Compared with HFD-fed *NLRP3*^*−/−*^*Ldlr*^*−/−*^ mice, APN 50 treatment could not further reduce hepatic lipid accumulation (Fig. 6C,D). Consistent with these results, APN treatment did not further improve NAFLD indicators in *NLRP3*^*−/−*^*Ldlr*^*−/−*^ mice (data not shown). Atherosclerosis lesion analysis showed no obvious differences between the HFD and HFD + APN 50 group in *NLRP3*^*−/−*^*Ldlr*^*−/−*^ mice (Fig. 6E,F).

## Discussion

Both NAFLD and atherosclerosis are inflammatory diseases with significant morbidity and mortality^26,27^. A better understanding of the mechanisms underlying NAFLD and atherosclerosis is important for developing more effective diagnostic and therapeutic strategies. In the present study, we evaluated the effect of APN on NAFLD and atherosclerosis using *Ldlr*^*−/−*^ and *NLRP3*^*−/−*^*Ldlr*^*−/−*^ mice fed with a HFD. APN is a natural flavonoid, and its anti-inflammation of the APN has been well-established^28,29^. However, the effect of APN on NAFLD and atherosclerosis, and the underlying mechanism, remain elusive. The present study demonstrated that APN had a protective effect on the development of NAFLD and atherosclerosis and that the mechanism may involve the inhibition of the NLRP3/NF-κB signaling pathway.

APN is one of the most widespread flavonoids in vegetables (parsley, celery, onions), fruits (oranges), herbs (chamomile, thyme, oregano, basil), and plant-based beverages (tea, beer and wine)^30^. A huge number of studies have reported the antioxidant and anti-inflammatory properties of APN^31,32^. In addition, antiatherogenic^33,33^ and anti-apoptotic^34^, as well as the protective effects against NAFLD, cardiac hypertrophy and hypertension^35^ have been reported. In our study, the protective effect of APN on NAFLD was shown in HE and Oil red O staining of liver. Histological analysis revealed significantly less hepatic lipid deposition in APN supplemented mice when compared with control mice. In APN-treated atherosclerosis mice, the inflammatory cytokines IL-1β and IL-18 were significantly decreased. Furthermore, APN attenuated atherosclerotic lesion. We further confirmed that NLRP3 deficiency did not alleviate NAFLD and atherosclerosis after APNtreatment. Taken together, our data demonstrated that APN protects from NAFLD and atherosclerosis through the inhibition of NLRP3 inflammasome.

The role of inflammasome activation in NAFLD has received significant attention in recent years^36,37^. The NLRP3 inflammasome is primarily activated by inflammatory stimuli. The initiating step involves a signal in which inflammatory stimuli are recognized by toll-like receptors, leading to the activation of NF-κB. The activation of NF-κB upregulates inactive NLRP3. Then, the activation of NLRP3 triggers the transformation of procaspase-1 to caspase-1, as well as the production and secretion of mature IL-1β and IL-18^38,39^. Our results demonstrated that HFD enhanced the mRNA expression levels of inflammation-related genes (F4/80, MCP-1, TNF-α, IL-6, NLRP3, NF-κB, TGF-β1, IL-1β and IL-18). As expected, in the HFD-induced atherosclerotic *Ldlr*^*−/−*^ mouse model, IL-1β and IL-18 levels in arteries is significantly increased. In vivo experiments revealed that APN significantly downregulated the expression of inflammation related genes and protein levels. In vitro experiments, showed that APN attenuated lipid deposition in OA-stimulated HepG2 cells, in which NLRP3/ NF-κB signaling was inactivated significantly. In this study, we not only found that APN improved dyslipidemia, but also showed that APN alleviated inflammation and lipid accumulation by inhibiting the NLRP3/ NF-κB signaling pathway.

In conclusion, APN ameliorated NAFLD and atherosclerosis, and the underlying mechanism involved the inhibition of inflammation and NLRP3 activation. These findings indicated that APN had therapeutic potential in the prevention of NAFLD and atherosclerosis. Therefore, APN supplementation may be considered a potential prevention strategy for NAFLD and atherosclerosis.

## Supplementary Information

Supplementary Information.

## Data Availability

The datasets generated during and/or analyzed during the current study are available from the corresponding author on reasonable request.
