# Supplementary figures and images for: Apigenin attenuates atherosclerosis and non-alcoholic fatty liver disease through inhibition of NLRP3 inflammasome in mice
Source: Sci Rep. 2023 May 17;13:7996. doi: 10.1038/s41598-023-34654-2 (PMC10192453; doi:10.1038/s41598-023-34654-2)

Figure 4B.

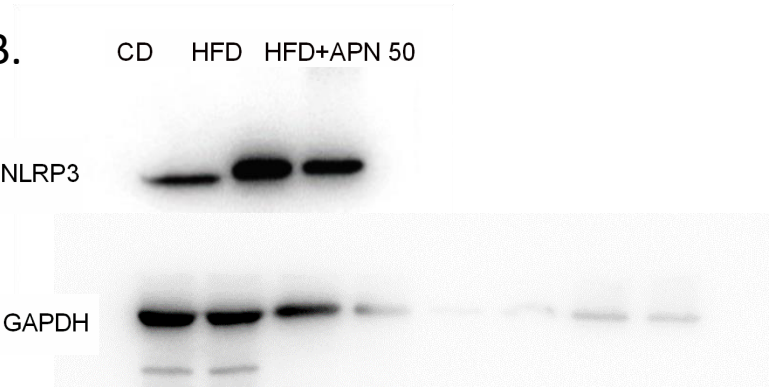

Figure 4C.

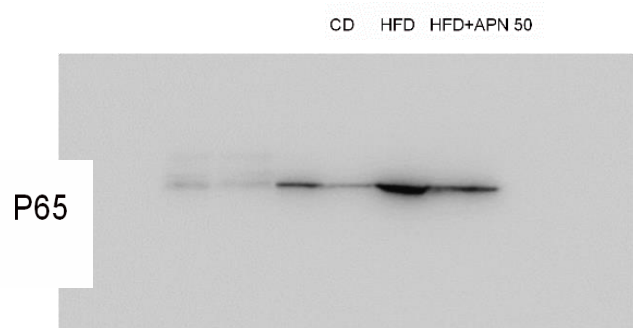

Figure 5D.

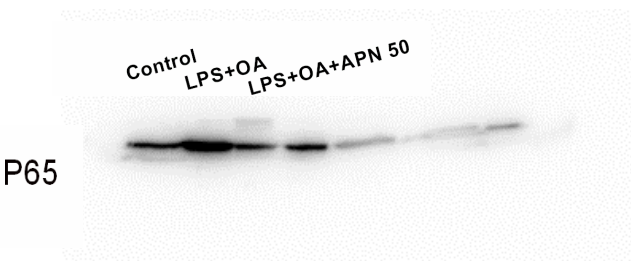

Figure 4C.

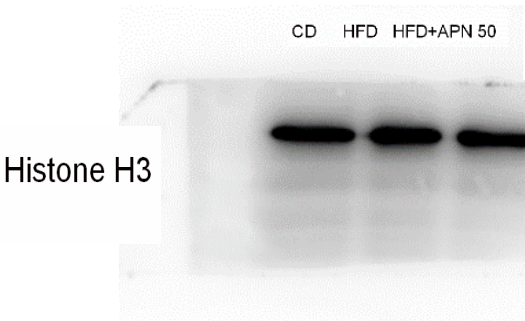

Figure 5D.

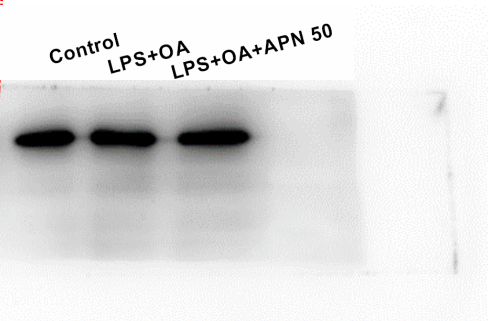

Control  
LPS+OA  
LPS+OA+APN 50

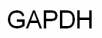

Supplement: Supplementary file 1 — Supplementary Information. [file 41598_2023_34654_MOESM1_ESM.pdf]
